# Contrastive and Transfer Learning for Aligned Multimodal Neuroimaging Classification of Autism Spectrum Disorder

**DOI:** 10.3390/jimaging12070328

**Published:** 2026-07-20

**Authors:** Raja Vavekanand, Ganesh Kumar, Muhammad Moazzam Jawaid, Shafiya Qadeer Memon, Teerath Kumar

**Affiliations:** 1 Department of Information Technology, Benazir Bhutto Shaheed University Lyari, Karachi 75660, Sindh, Pakistan; 2 Department of Computing, Universiti Teknologi PETRONAS, Seri Iskandar 32610, Malaysia; ganesh.kumar@utp.edu.my; 3 School of Computer Science and Informatics, De Montfort University, Leicester LE1 9BH, UK; 4 Department of Software Engineering, Mehran University of Engineering and Technology, Jamshoro 76062, Sindh, Pakistan; shafiya.memon@faculty.muet.edu.pk; 5 School of Computing, Atlantic Technological University, F94 DV52 Letterkenny, Ireland; teerathkumar.menghwar@atu.ie

**Keywords:** Autism Spectrum Disorder, multimodal classification, contrastive learning, transfer learning, neuroimaging

## Abstract

Autism Spectrum Disorder (ASD) assessment remains challenging because behavioural instruments are partly observer-dependent and neuroimaging data are heterogeneous. This paper presents FAA (Fuse After Aligned), which is a multimodal classification framework that combines transfer learning for structural MRI (sMRI) representation learning with a contrastive objective for the pre-fusion alignment of sMRI and resting-state functional MRI-derived functional connectivity (FC) features. Evaluation was restricted to the single-site ABIDE-I New York University subset comprising 75 participants with ASD and 98 typically developing controls. Under the reported five-fold internal cross-validation protocol, FAA achieved a mean accuracy of 92.6% compared with 87.4% for naive fusion and 90.9% for the sMRI-only baseline. Ablation analyses indicate that adding the contrastive objective is associated with improved classification performance and that ResNet-18 outperforms the evaluated ViT-16 configurations in this small-sample setting. These findings support the methodological value of pre-fusion feature alignment within the evaluated cohort. The framework offers a robust, computationally efficient, and clinically viable approach for objective ASD diagnosis with strong potential for generalisation to multi-site neuroimaging applications.

## 1. Introduction

ASD is a complex neurodevelopmental disorder defined by persistent challenges in social communication and interaction as well as restricted and repetitive patterns of behaviour, interests, and activities [1]. ASD is also associated with heterogeneous eating, gastrointestinal, and microbiome-related comorbidities [2]. It generally first becomes apparent in early childhood and continues throughout life; estimates are around 1 in 54 children. ASD has an economic cost, and in the United States, it costs more than USD $1.2 million per person for a lifetime of ASD [3]. The diagnosis of ASD is currently made based on observations of behaviour and use of standardised questionnaires and scales, including the Autism Diagnostic Observation Schedule (ADOS) and Autism Diagnostic Interview-Revised (ADI-R) [4,5,6]. The methods are, however, highly subjective, time-intensive, and demand specific clinical expertise, which is scarce [6]. This diagnostic problem has stimulated the search for objective, biomarker-based methods which could offer a more reliable and accessible diagnostic support.

Neuroimaging methods, especially structural and functional magnetic resonance imaging (sMRI and fMRI), have proven to be excellent methods for detecting neurological markers of ASD [7,8]. The results from sMRI studies in ASD patients have shown structural abnormalities such as alterations in gray matter volume [9,10], in cortical thickness [11] as well as in white matter organisation [12]. Recently, resting-state functional MRI (rs-fMRI) studies have shown abnormal functional connectivity patterns in ASD brains in various brain networks, which is in line with the theories of “underconnectivity” [12] and “overconnectivity” [13] of the ASD brain. Structural and functional data are complementary; this has led to an increased interest in multimodal approaches to ASD classification [14]. Unimodal approaches that rely only on sMRI [15,16] or rs-fMRI [12,17,18] have been successful, but they only incorporate some dimensions of the disorder’s complex neurobiology. Theoretically, multimodal integration provides a more complete characterisation of the ASD pathology based on the integration of structural abnormalities with functional network alterations [9].

There are, however, two main problems with the current multimodal methods. First, the naive mixing of features from different modalities may not be able to deal with the modality heterogeneity of the features [19]. The two types of data, sMRI and rs-fMRI, are measured in two different domains: structural features measure anatomical properties, and functional connectivity measures temporal correlations in neural activity. While it is possible to simply concatenate these features without alignment and achieve suboptimal fusion results, these results can be seen in naive fusion approaches [20]. Second, because of the scarcity of labeled neuroimaging data, deep learning models for medical image analysis are especially susceptible to overfitting, which further hampers their generalisability across the target population of individuals with autism spectrum disorder and the general population of children [15,20].

Training complex architectures from scratch is difficult when labelled neuroimaging cohorts are small. Transfer learning can reduce this constraint for sMRI representation learning, and transformer-based sMRI classification has also been explored in neurological imaging [21], although the relative behaviour of convolutional and transformer backbones remains sensitive to dataset size and pre-training strategy. Cross-site variation, limited interpretability, and heterogeneous modality distributions further restrict the evidential scope of neuroimaging classifiers. FAA (Fuse After Aligned) addresses feature heterogeneity and data scarcity through contrastive pre-fusion alignment and transfer learning. There are three principal contributions:

- A CLIP-inspired contrastive objective [22] that maps paired sMRI-derived anatomical representations and rs-fMRI-derived FC representations into a common embedding space before additive fusion.
- A controlled comparison of ResNet-18 and ViT-16 under the same single-site, small-sample experimental setting, including pre-training, fine-tuning, and contrastive-loss ablations.
- An internal evaluation on the ABIDE-I NYU subset, yielding a mean accuracy of 92.6% under the reported five-fold protocol, together with explicit limitations concerning external generalisability, fold-wise stability, advanced fusion baselines, and biological interpretability.

The rest of this paper is organised as follows: Section 2 provides a literature review. The FAA methodology is described in Section 3. The experimental results and ablation studies and comparison are presented in Section 4. Section 5 discusses the implications of the findings, model limitations, and future directions. Lastly, Section 6 brings the paper to a close.

## 2. Literature Review

### 2.1. Unimodal Neuroimaging Classification

MRI-based machine learning has been investigated as a source of quantitative support for ASD assessment, but systematic and umbrella reviews consistently identify small cohorts, site-related variability, inconsistent biomarkers, and limited external validation as major barriers [23,24,25]. Unimodal rs-fMRI studies commonly represent functional organisation through connectivity matrices or graphs. Representative approaches include multi-view spectral graph convolution [26], relational graph attention [27], residual graph transformers [28], and multi-atlas ensemble learning [29]. These methods demonstrate that FC contains discriminative information, but they do not exploit paired structural and functional measurements within a common subject-level representation.

### 2.2. Multimodal Fusion of Structural and Functional MRI

Combining sMRI-derived anatomy with rs-fMRI-derived functional organisation can provide complementary information [30]. Existing multimodal studies have examined deep multimodal networks, 3D convolutional or transformer models, joint fusion, and attention-pruned graph architectures [31,32]. Most reported strategies use early fusion, late fusion, concatenation, voting, or attention [11]. Because sMRI and FC encode different biological quantities and have different dimensional structures [33], direct fusion can amplify modality imbalance or redundant features. An explicit subject-paired alignment of structural and functional embeddings before fusion remains comparatively underexplored.

### 2.3. Contrastive Learning, Transfer Learning, and Explainability

Contrastive objectives have improved neuroimaging representation learning in graph-based and self-supervised settings, including A-GCL [34] and unsupervised contrastive graph learning [35]. These approaches primarily operate within FC or graph representations rather than aligning sMRI with FC. Transfer learning is also relevant because ASD neuroimaging cohorts are typically much smaller than the natural-image datasets used to train modern backbones. Convolutional networks provide locality and translation-related inductive biases, whereas vision transformers generally depend more strongly on dataset scale and pre-training. Explainability remains an additional requirement for biological interpretation; attribution analyses can identify anatomical regions or functional connections that influence model outputs [36].

### 2.4. Research Gap and Study Scope

FAA evaluates two questions within a single-site internal-validation setting: whether a contrastive objective added before fusion is associated with improved classification and how ResNet-18 compares with ViT-16 under the same small-sample conditions. The current evidence does not isolate pair-specific alignment from generic auxiliary-loss regularisation because a shuffled-pairing control was not performed. It also does not establish superiority over gated or cross-attention fusion under identical splits, and it does not provide regional attribution or external multi-site validation. Table 1 therefore positions FAA by architectural characteristics rather than by claiming directly comparable superiority across studies.

## 3. Methodology

**Figure 1:**
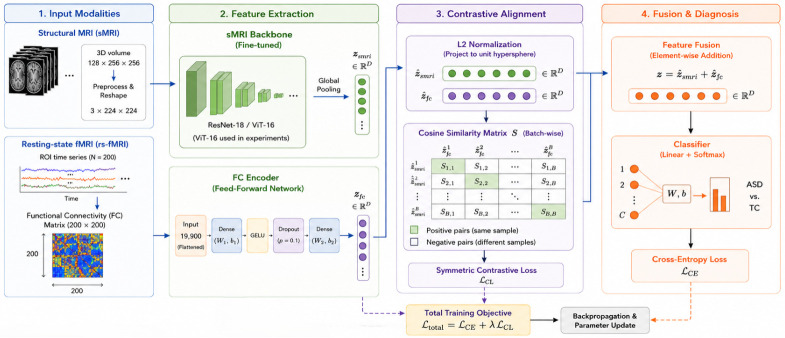
Overall architecture of the proposed FAA framework. The framework comprises three main components: (1) feature extraction using a fine-tuned ResNet-18 for sMRI and a Feed-Forward Network (FFN) for functional connectivity (FC) features; (2) a contrastive learning module that aligns sMRI and FC features in a shared semantic space using a CLIP-inspired objective; and (3) a fusion and classification module that combines aligned features for final ASD versus TC classification. The contrastive loss $L_{CL}$ and classification loss $L_{CE}$ are jointly optimised during training.

The proposed FAA (Fuse After Aligned) framework integrates structural magnetic resonance imaging (sMRI) and resting-state functional magnetic resonance imaging (rs-fMRI) data for ASD versus typical control (TC) classification. The architecture, illustrated in Figure 1, consists of three primary stages: (1) modality-specific feature extraction, (2) contrastive feature alignment, and (3) feature fusion and classification. Each component is described in detail in the following subsections.

### 3.1. Data Preprocessing

#### 3.1.1. sMRI Data Preprocessing

The structural branch receives CAT12-derived grey-matter probability maps rather than unprocessed T1-weighted images. Each T1-weighted scan is corrected for intensity inhomogeneity, skull-stripped, segmented into tissue classes with CAT12 for SPM12, spatially normalised, modulated, and smoothed using an 8 mm full-width-at-half-maximum Gaussian kernel. The final grey-matter map is represented as $X_{\mathrm{GM}}\in R^{128\times 256\times 256}$, where the 128 axial slices are treated as input channels. Fixed intensity scaling is applied before network input.

**Figure 2:**
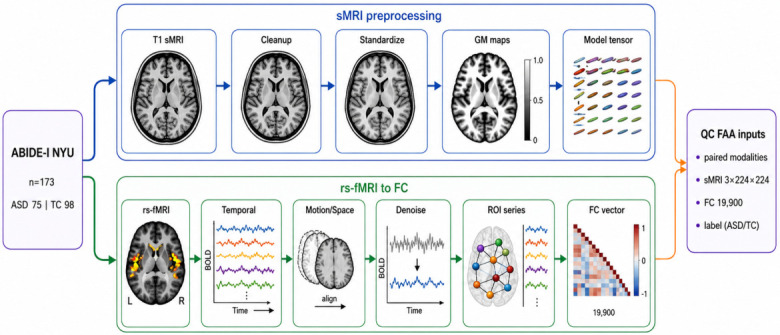
Data preprocessing pipeline before FAA feature extraction, contrastive alignment, additive fusion, and ASD-versus-TC classification.

Compatibility with ImageNet-pre-trained backbones is obtained through a learnable two-dimensional convolution with 128 input channels, 3 output channels, a 33×33 kernel, and no padding. This operation converts $X_{\mathrm{GM}}$ into ${\hat{X}}_{\mathrm{smri}}\in R^{3\times 224\times 224}$ before ResNet-18 or ViT-16 processing. Consequently, the network input is the transformed CAT12 grey-matter map rather than the original T1-weighted volume. Figure 2 summarises the harmonised pipeline.

#### 3.1.2. rs-fMRI Data Preprocessing

Resting-state fMRI data undergo a comprehensive preprocessing pipeline to remove nuisance signals and derive reliable functional connectivity measures. The pipeline includes the following sequential steps:

1. **Slice-timing correction**: Interleaved slice acquisition is corrected using sinc-interpolation to temporally align all slices to the middle slice.
2. **Motion correction**: Rigid-body realignment is performed to correct for head motion during scanning. Subjects with mean framewise displacement exceeding 0.5 mm are excluded from further analysis.
3. **Spatial normalisation**: Each functional volume is co-registered to the subject’s T1-weighted anatomical image and subsequently warped to MNI152 standard space.
4. **Spatial smoothing**: A Gaussian kernel with full-width at half-maximum (FWHM) of 6 mm is applied to improve the signal-to-noise ratio.
5. **Nuisance signal regression**: Signals from cerebrospinal fluid, white matter, and six head motion parameters are regressed out to remove non-neuronal contributions.
6. **Bandpass filtering**: A frequency filter (0.01–0.1 Hz) is applied to isolate low-frequency fluctuations of interest.

Following preprocessing, the mean time series is extracted from each of N=200 regions of interest defined by the CC200 functional atlas [37]. CC200 was selected because it is a data-driven whole-brain parcellation widely used in ABIDE analyses and provides an intermediate spatial resolution: 200 regions preserve distributed network information while limiting the upper-triangular FC representation to 19,900 pairwise connections. This dimensionality is tractable for the available sample size and the feed-forward FC branch. No alternative-atlas experiment was conducted; therefore, atlas dependence is acknowledged as a limitation. The resulting time-series matrix $T\in R^{T\times N}$, where *T* denotes the number of time points, forms the basis of FC computation.

### 3.2. Feature Extraction

#### 3.2.1. sMRI Feature Extraction with Transfer Learning

**Figure 3:**
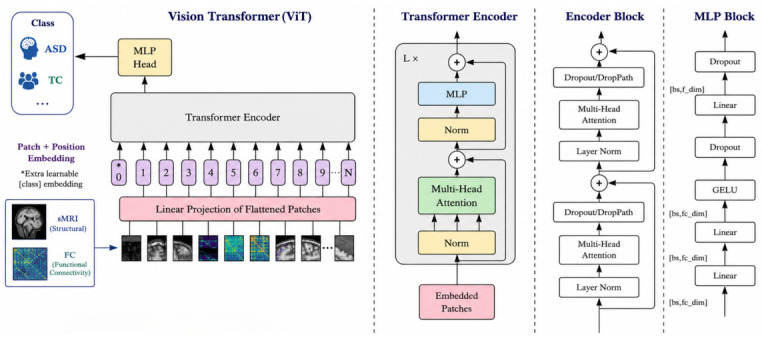
Architecture of the vision transformer (ViT-16) used in the experiments. The input image is divided into 16×16 patches, which are linearly projected and supplemented with positional embeddings. The resulting sequence is processed through a transformer encoder comprising multi-head self-attention and feed-forward layers. A classification token is prepended to the sequence, and the corresponding output is used as the image representation.

The sMRI feature extraction module employs a pre-trained convolutional neural network backbone to capture anatomical patterns discriminative of ASD. The processed sMRI volume ${\hat{X}}_{\mathrm{smri}}\in R^{3\times 224\times 224}$ is passed through the feature extraction network:

(1) $$z_{\mathrm{smri}}=\mathrm{Backbone}\left({\hat{X}}_{\mathrm{smri}}\right),\;z_{\mathrm{smri}}\in R^{D}$$

where Backbone(·) denotes either ResNet-18 or ViT-16, and *D* is the extracted feature dimension. The backbone is initialised with ImageNet-pre-trained weights and fine-tuned on sMRI data. ResNet-18 is used as the main backbone due to its efficiency, suitability for small medical datasets, and ability to capture local spatial brain patterns through residual learning. For comparison, ViT-16 is also evaluated by splitting images into 16×16 patches and processing them with a transformer encoder, as shown in Figure 3.

#### 3.2.2. rs-fMRI Feature Extraction via Functional Connectivity

Functional connectivity features are derived from the preprocessed rs-fMRI time series. For each subject, an FC matrix $C\in R^{N\times N}$ is constructed by computing the Pearson correlation coefficient between the time series of each pair of ROIs:

(2) $$C_{ij}=\frac{\sum_{t=1}^{T}\left(T_{it}-{\bar{T}}_{i}\right)\left(T_{jt}-{\bar{T}}_{j}\right)}{\sqrt{\sum_{t=1}^{T}{\left(T_{it}-{\bar{T}}_{i}\right)}^{2}}\sqrt{\sum_{t=1}^{T}{\left(T_{jt}-{\bar{T}}_{j}\right)}^{2}}}$$

where $T_{it}$ denotes the BOLD signal intensity at ROI *i* and time point *t*, and ${\bar{T}}_{i}$ represents the temporal mean of the signal at ROI *i*. The upper triangle of C and the main diagonal are removed to eliminate redundant information and self-correlations, respectively. The remaining elements are flattened into a feature vector $x_{\mathrm{fc}}\in R^{F}$, where

(3) $$F=\frac{N\times (N-1)}{2}=19,900$$

for N=200 ROIs.

**Figure 4:**
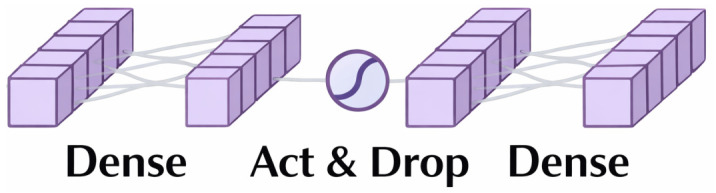
Schematic diagram of the Feed-Forward Network (FFN) architecture for rs-fMRI feature extraction.

The high-dimensional FC feature vector is processed through a Feed-Forward Network (FFN) with two linear layers, GELU activation, and Dropout regularisation. The FFN architecture, illustrated in Figure 4, projects $x_{\mathrm{fc}}$ into a compact representation:

(4) $$\begin{array}{cc} {\hat{x}}_{\mathrm{fc}} & =x_{\mathrm{fc}}W_{1}+b_{1},\;W_{1}\in R^{F\times D},\;b_{1}\in R^{D} \end{array}$$

(5) $$\begin{array}{cc} z_{\mathrm{fc}}^{\prime } & =\mathrm{GELU}({\hat{x}}_{\mathrm{fc}})\; \end{array}$$

(6) $$\begin{array}{cc} \;z_{\mathrm{fc}} & =z_{\mathrm{fc}}^{\prime }W_{2}+b_{2},\;W_{2}\in R^{D\times D},\;b_{2}\in R^{D} \end{array}$$

The output $z_{\mathrm{fc}}\in R^{D}$ represents the functional connectivity features in the same dimensionality as the sMRI features, enabling subsequent alignment operations.

### 3.3. Contrastive Feature Alignment

To align the modality-specific features $z_{\mathrm{fc}}$ and $z_{\mathrm{smri}}$ into a shared semantic space, a contrastive learning objective is employed. The alignment process consists of three steps: normalisation, similarity computation, and contrastive loss optimisation.

#### 3.3.1. L2 Normalisation

The feature vectors from both modalities are first subjected to L2 normalisation to project them onto a unit hypersphere:

(7) $$\begin{array}{cc} {\hat{z}}_{\mathrm{fc}} & =\frac{z_{\mathrm{fc}}}{∥z_{\mathrm{fc}}{∥}_{2}} \end{array}$$

(8) $$\begin{array}{cc} {\hat{z}}_{\mathrm{smri}} & =\frac{z_{\mathrm{smri}}}{∥z_{\mathrm{smri}}{∥}_{2}} \end{array}$$

This normalisation ensures that the similarity metric is based on angular distances rather than magnitude differences, which is particularly important when features from different modalities exhibit different scales.

#### 3.3.2. Similarity Computation

Given a batch of *B* subjects, the normalised feature matrices ${\hat{Z}}_{\mathrm{fc}}\in R^{B\times D}$ and ${\hat{Z}}_{\mathrm{smri}}\in R^{B\times D}$ are constructed. A batch-wise cosine similarity matrix $S\in R^{B\times B}$ is computed as

(9) $$S={\hat{Z}}_{\mathrm{fc}}\cdot {\hat{Z}}_{\mathrm{smri}}^{⊤}$$

Each element $S_{ij}$ represents the cosine similarity between the FC features of subject *i* and the sMRI features of subject *j*. The diagonal elements $S_{ii}$ correspond to the similarity between the paired modalities of the same subject.

#### 3.3.3. Contrastive Loss

The symmetric contrastive loss $L_{\mathrm{CL}}$ is defined as

(10) $$L_{\mathrm{CL}}=-\frac{1}{2B}\sum_{i=1}^{B}\left[\log\frac{\exp(S_{ii}/\tau )}{\sum_{j=1}^{B}\exp\left(S_{ij}/\tau \right)}+\log\frac{\exp(S_{ii}/\tau )}{\sum_{j=1}^{B}\exp\left(S_{ji}/\tau \right)}\right]$$

where τ is a temperature hyperparameter that controls the sharpness of the softmax distribution, and *B* is the batch size. The first term encourages the FC features of each subject to be similar to their corresponding sMRI features (positive pairs), while the second term symmetrically enforces the same relationship.

The contrastive objective is intended to increase the similarity between paired structural and functional representations while separating non-matching samples within a batch. It may also act as an auxiliary regulariser. Because the current ablation compares only training with and without the additional objective, the observed gain cannot be attributed exclusively to subject-pair correspondence. A shuffled-pairing control, in which structural and functional samples are deliberately mismatched during training, is required to distinguish pair-specific alignment from generic regularisation.

### 3.4. Feature Fusion and Classification

#### 3.4.1. Feature Fusion

Following contrastive alignment, the normalised features from both modalities are fused via element-wise addition:

(11) $$z={\hat{z}}_{\mathrm{fc}}+{\hat{z}}_{\mathrm{smri}},\;z\in R^{D}$$

This additive fusion strategy is selected for its simplicity and effectiveness. Since the features have been aligned in the shared semantic space, addition serves as a natural aggregation operation that preserves the information content of both modalities without introducing additional learnable parameters. Alternative fusion strategies, including concatenation and weighted summation.

#### 3.4.2. Classification

The fused representation z is passed through a linear classification layer to produce the final prediction:

(12) $$\hat{y}=\mathrm{Softmax}\left(zW_{\mathrm{cls}}+b_{\mathrm{cls}}\right)$$

where $W_{\mathrm{cls}}\in R^{D\times 2}$ and $b_{\mathrm{cls}}\in R^{2}$ are the weight matrix and bias vector of the classification layer, respectively. The output $\hat{y}\in R^{2}$ represents the predicted probability distribution over the two classes (ASD and TC). The predicted class is determined by

(13) $$\hat{c}=\arg\max_{k\in \{0,1\}}{\hat{y}}_{k}$$

### 3.5. Overall Optimisation Objective

The FAA framework is trained end-to-end using a joint optimisation objective that combines the cross-entropy classification loss with the contrastive alignment loss. The total loss function is defined as

(14) $$L_{\mathrm{total}}=L_{\mathrm{CE}}+\lambda L_{\mathrm{CL}}$$

where $L_{\mathrm{CE}}$ is the cross-entropy classification loss:

(15) $$L_{\mathrm{CE}}=-\frac{1}{B}\sum_{i=1}^{B}\sum_{k=0}^{1}y_{ik}\log\left({\hat{y}}_{ik}\right)$$

and λ is a weighting hyperparameter that balances the contributions of the two loss components. The hyperparameter λ is set to 1 in the primary experiments with ablation studies examining the effect of varying this parameter.

The complete training procedure is summarised in Algorithm 1. The model is trained using the AdamW optimiser with a cosine annealing learning rate schedule. Early stopping is employed with a patience of 4 epochs to prevent overfitting.
**Algorithm 1** End-to-End Training Procedure for the FAA Framework**Require:** Training dataset $D={\left\{\left(X_{\mathrm{smri}}^{\left(i\right)},X_{\mathrm{fc}}^{\left(i\right)},y^{\left(i\right)}\right)\right\}}_{i=1}^{N}$, batch size *B*, temperature τ, loss weight λ, learning rate η**Ensure:** Trained model parameters θ1:Initialise the ResNet-18 backbone with ImageNet-pretrained weights2:Initialise the FFN parameters randomly3:Initialise the classifier parameters randomly4:**while** the training process has not converged **do**5:     Sample a mini-batch ${\left\{\left(X_{\mathrm{smri}}^{\left(i\right)},X_{\mathrm{fc}}^{\left(i\right)},y^{\left(i\right)}\right)\right\}}_{i=1}^{B}$ from D**Feature Extraction**6:     Extract sMRI features:$$Z_{\mathrm{smri}}=\mathrm{Backbone}\left(X_{\mathrm{smri}}\right)$$7:     Extract FC features:$$Z_{\mathrm{fc}}=\mathrm{FFN}\left(X_{\mathrm{fc}}\right)$$**Contrastive Alignment**8:     Apply L2 normalisation:$${\hat{Z}}_{\mathrm{smri}}=L2\mathrm{Norm}\left(Z_{\mathrm{smri}}\right),\;{\hat{Z}}_{\mathrm{fc}}=L2\mathrm{Norm}\left(Z_{\mathrm{fc}}\right)$$9:     Compute the cross-modal similarity matrix:$$S={\hat{Z}}_{\mathrm{fc}}{\hat{Z}}_{\mathrm{smri}}^{⊤}$$10:     Compute the bidirectional contrastive loss:$$L_{\mathrm{CL}}=-\frac{1}{2B}\sum_{i=1}^{B}\left[\log\frac{\exp(S_{ii}/\tau )}{\sum_{j=1}^{B}\exp\left(S_{ij}/\tau \right)}+\log\frac{\exp(S_{ii}/\tau )}{\sum_{j=1}^{B}\exp\left(S_{ji}/\tau \right)}\right]$$**Fusion and Classification**11:     Fuse the aligned modality representations:$$Z={\hat{Z}}_{\mathrm{fc}}+{\hat{Z}}_{\mathrm{smri}}$$12:     Generate class predictions:$$\hat{Y}=\mathrm{Softmax}\left(ZW_{\mathrm{cls}}+b_{\mathrm{cls}}\right)$$13:     Compute the classification loss:$$L_{\mathrm{CE}}=-\frac{1}{B}\sum_{i=1}^{B}\sum_{k}y_{ik}\log\left({\hat{y}}_{ik}\right)$$**Optimisation**14:     Compute the total training objective:$$L_{\mathrm{total}}=L_{\mathrm{CE}}+\lambda L_{\mathrm{CL}}$$15:     Update all trainable parameters:$$\theta \leftarrow \theta -\eta \nabla_{\theta }L_{\mathrm{total}}$$16:**end while**17:**return** trained model parameters θ

## 4. Experiments and Results

This section presents the experimental evaluation of the proposed FAA framework. The experimental setup is first described, which is followed by comprehensive results covering the overall classification performance, transfer learning analysis, contrastive learning effectiveness, hyperparameter sensitivity, and computational efficiency. Statistical analyses and ablation studies are provided to validate the contributions of each architectural component.

### 4.1. Dataset Description and Experimental Setup

#### 4.1.1. ABIDE-I Dataset

The proposed framework is evaluated on a subset of the Autism Brain Imaging Data Exchange-I (ABIDE-I) dataset [38]. ABIDE-I is a multi-site neuroimaging database comprising structural and resting-state functional MRI data from over 1000 subjects across 17 international sites. The subset employed in this paper consists of 173 subjects from the New York University (NYU) site, including the following:

- **ASD group**: 75 subjects diagnosed with Autism Spectrum Disorder according to DSM-IV criteria, confirmed through the Autism Diagnostic Observation Schedule (ADOS) and the Autism Diagnostic Interview-Revised (ADI-R).
- **Typical Control (TC) group**: 98 age- and sex-matched typically developing individuals with no history of psychiatric or neurological disorders.

Table 2 presents the demographic characteristics of the study population. No statistically significant differences are observed between the ASD and TC groups with respect to age (p=0.47, two-sample *t*-test) or sex distribution (p=0.31, chi-square test), ensuring that observed performance differences are not attributable to demographic confounding.

The sMRI and rs-fMRI data follow the pipeline described in Section 3.1. The sMRI data are intensity-corrected, skull-stripped, CAT12-segmented, normalised, modulated, and smoothed with an 8 mm FWHM kernel. The resulting grey-matter maps (128×256×256) are converted to 3×224×224 before ResNet-18 input. The rs-fMRI data are preprocessed in DPABI using time-point removal, slice-timing and motion correction, Friston-24 regression, MNI152 normalisation, 6 mm smoothing, 0.01–0.1 Hz filtering, and nuisance regression. Subjects with mean framewise displacement above 0.5 mm are excluded.

#### 4.1.2. Implementation and Evaluation Protocol

All models are implemented in PyTorch 1.13.0 and trained on an NVIDIA RTX 3090 GPU using AdamW with a learning rate of $1\times 10^{-5}$, weight decay of $1\times 10^{-4}$, batch size 16, and a maximum of 24 epochs. Early stopping is applied after four epochs without improvement. Evaluation uses shuffled five-fold cross-validation with four folds for training and one fold for both early stopping and performance assessment. Therefore, the results represent internal-validation estimates and may be optimistic because no independent test set is used. Reported metrics are averaged across the five held-out folds with 95% bootstrap intervals calculated from 1000 resamples.

### 4.2. Overall Classification Performance

Table 3 presents the comprehensive comparison of different classification methods on the ABIDE-I NYU dataset. The following methods are evaluated:

- **FC-only**: functional connectivity features processed through the FFN backbone.
- **sMRI-only**: structural MRI features extracted using ResNet-18 pre-trained on ImageNet and fine-tuned on the target dataset.
- **sMRI+FC (Naive Fusion)**: simple additive fusion of sMRI and FC features without contrastive alignment.
- **FAA (Proposed)**: the complete framework with contrastive alignment followed by additive fusion.

Within the reported internal-validation protocol, the sMRI-only model achieves a mean accuracy of 90.9% compared with 76.5% for FC-only. FC-only yields low ASD accuracy (45.0%) despite a reported TC accuracy of 100.0%, indicating strong class-asymmetric behaviour. The sMRI-only result is less asymmetric (79.2% ASD; 98.2% TC). Naive additive fusion reaches 87.4%, whereas FAA reaches 92.6%, corresponding to mean differences of 5.2 percentage points relative to naive fusion and 1.7 percentage points relative to sMRI-only. The repeated 100.0% TC values must be interpreted cautiously because the cohort is small, the reporting is aggregate, and the early-stopping fold is also used for evaluation. They do not establish stable clinical specificity.

**Figure 5:**
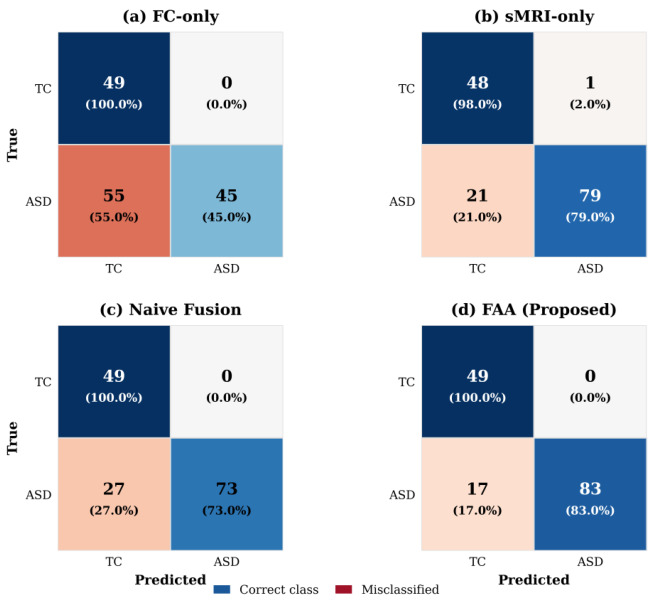
Aggregate confusion-matrix summaries for the evaluated methods under internal cross-validation.

FC-only misclassifies 55.0% of ASD cases as TC, demonstrating pronounced class asymmetry in this cohort. sMRI-only reports 79.2% ASD accuracy and 98.2% TC accuracy, while naive fusion reports 72.5% ASD accuracy. Figure 5 summarises the aggregate class-conditional results. FAA reports 82.7% ASD accuracy and 100.0% TC accuracy.

### 4.3. Impact of Transfer Learning and Backbone Selection

Backbone architectures, ResNet-18 and ViT-16, are compared and evaluated under various training configurations, as listed in Table 4. ResNet-18 is always a better performer than ViT-16, especially in terms of accuracy of ASD detection in the fully optimised setup (79.2% vs. 22.8%), indicating CNNs perform better in limited data medical imaging. Clearly, pre-training on ImageNet has a positive impact on ResNet-18 accuracy (from 84.0% to 90.9%), and freezing without fine-tuning leads to only 88.4% accuracy, necessitating the need for domain adaptation. To conclude, ViT-16 seems to be underperforming with 0.0% ASD detection, which is likely due to its reliance on massive amounts of data and its weaker local spatial inductive bias.

**Figure 6:**
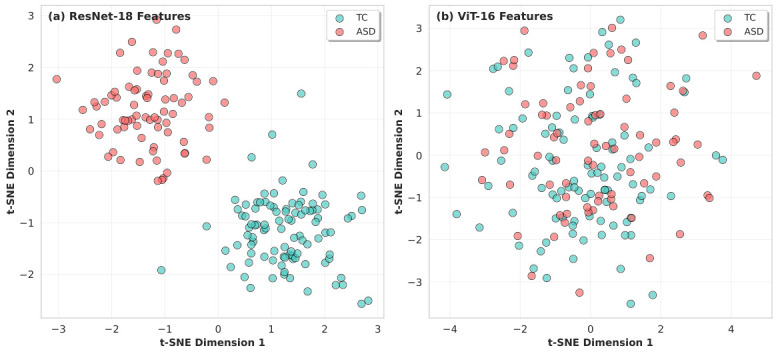
The t-SNE (t-distributed Stochastic Neighbour Embedding) visualisations of sMRI feature embeddings learned by ResNet-18 (**a**) and ViT-16 (**b**). The ResNet-18 is more clearly divided between ASD and TC classes with little overlap in the boundaries of the clusters. In contrast, the ViT-16 features show substantial intermingling of the two classes, explaining the inferior classification performance.

The learned representations are analysed qualitatively by projecting the embeddings of the sMRI into the t-SNE space, as seen in Figure 6 for both ResNet-18 and ViT-16. The separation of ASD and TC is enhanced with smaller cluster overlap in ResNet-18, suggesting better discriminative structural representations. ViT-16 exhibits a high degree of class mixing, however, and it is likely due to this factor that the classification performance is weak. This implies that CNN-based local spatial inductive bias is more suitable for small sample structural MRI analysis, and transformer-based global attention maybe needs to be based on larger datasets.

### 4.4. Effectiveness of Contrastive Feature Alignment

#### 4.4.1. Ablation Study on Contrastive Learning

For the pre-trained and fine-tuned ResNet-18 configuration, adding the contrastive objective increases mean accuracy from 87.4% to 92.6% (+5.2 percentage points). Table 5 compares training with and without this objective across the evaluated backbone settings. The direction and magnitude of the change vary by configuration, including degradation for some non-pre-trained settings, which indicates that the auxiliary objective is not uniformly beneficial. A shuffled-pairing control is therefore necessary for a causal interpretation of alignment.

#### 4.4.2. Visualisation of Feature Alignment

**Figure 7:**
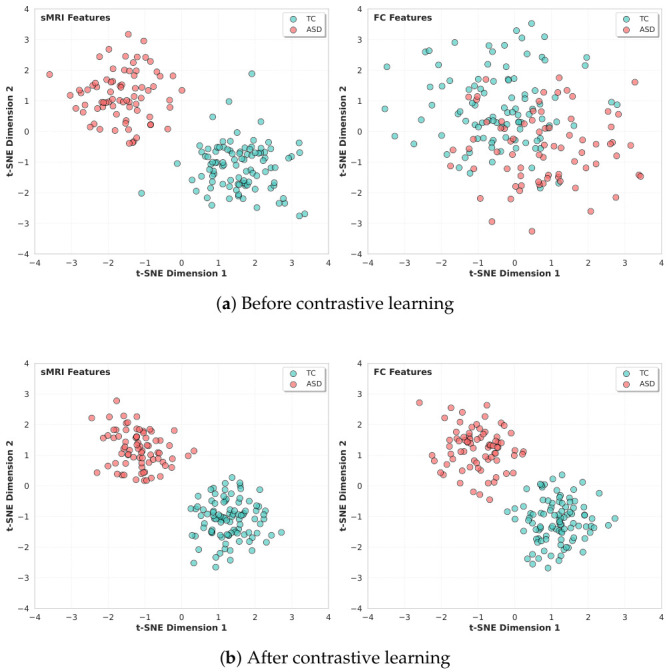
t-SNE visualisation of feature distributions. Before contrastive learning, sMRI and FC features occupy separate and unaligned spaces with different cluster structures. After contrastive learning, features from both modalities are aligned in a shared semantic space, showing improved class separation and more consistent clustering patterns across modalities.

Before alignment, the sMRI and FC projections occupy different regions with moderate ASD–TC separation in sMRI and greater overlap in FC. After training with the contrastive objective, the projected modalities appear more closely organised and the classes appear more separated. Because t-SNE is qualitative and sensitive to projection settings, this visualisation is supportive rather than causal evidence of subject-paired alignment. Figure 7 provides a qualitative two-dimensional projection of the learned representations before and after inclusion of the contrastive objective.

#### 4.4.3. Quantitative Alignment Metrics

To quantitatively assess the effectiveness of contrastive alignment, the Centered Kernel Alignment (CKA) between the feature spaces is computed. CKA measures the similarity between representations learned by different models with higher values indicating greater alignment (Table 6).

Contrastive learning improves CKA similarity across all configurations with the highest alignment score obtained in the pre-trained and fine-tuned setup (0.78). The CKA gains range from 0.39 to 0.47, confirming a substantial transformation of the multimodal feature space and supporting the t-SNE observations. The correspondence between higher CKA scores and better classification performance further highlights the importance of semantic feature alignment for effective multimodal fusion.

### 4.5. Analysis of Hyperparameters

#### 4.5.1. Temperature Parameter Sensitivity

The temperature parameter τ in the contrastive loss controls the sharpness of the similarity distribution and critically affects feature alignment. Table 7 presents the impact of τ on classification performance.

The optimal performance of ResNet-18 occurs at τ=1.0 with a stable and optimal scaling of contrastive similarity in all of the configurations. Compared to this, the performance of ViT-16 is better at a higher τ=4.0, highlighting the need for softer similarity distributions because of weaker representations of features. It also has more variability in performance over τ values. The results demonstrate that the optimal contrastive temperature is architecture-dependent and needs to be tuned by architecture.

#### 4.5.2. Effect of Loss Weighting Parameter λ

**Figure 8:**
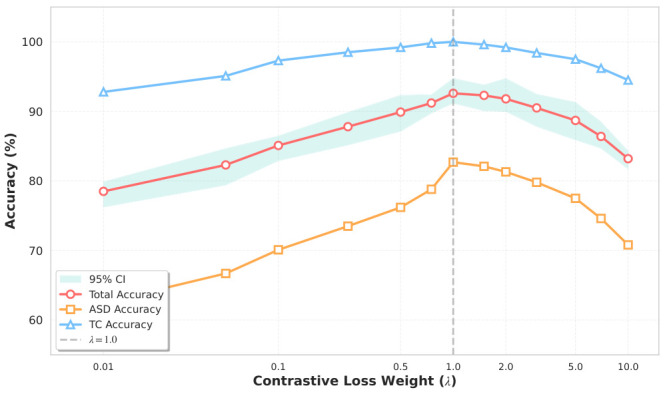
Effect of the contrastive loss weight λ on classification accuracy. The optimal performance is achieved at λ=1.0 with performance degrading at both lower and higher values. The performance plateau between λ=0.5 and λ=2.0 indicates robustness to moderate variations in the loss weighting.

The contribution of the contrastive loss to the total objective is controlled by the weighting parameter λ. The performance as a function of λ is shown in Figure 8. The optimal performance is achieved at the value of λ=1.0 with the total accuracy of 92.6%, suggesting a tradeoff between the contribution of the contrastive alignment and classification objectives. When λ<0.1, it is found that the performance drops, as the alignment signal is too weak to project multimodal features into a common semantic space.

### 4.6. Optimal Configuration Analysis

From the extensive experimental investigation, the optimum operational parameters for each of the backbones was determined and is summarised in Table 8. Pre-trained and fine-tuned ResNet-18 with contrastive learning (92.6%, 82.7%, 100.0%) was the best performing model. This setup has the best tradeoff of discriminative representation learning and data efficiency. The best performance was achieved with frozen pre-trained features with contrastive learning (88.7% accuracy), demonstrating that functional connectivity information partially mitigates for less robust image-based feature extraction for ViT-16 (Table 8). The frozen pre-trained ResNet-18 configuration continued to be competitive with an accuracy performance of 89.7%. However, in the absence of transfer learning, both backbones performed worse when trained from scratch, emphasising the need for transfer learning with small medical imaging datasets.

**Figure 9:**
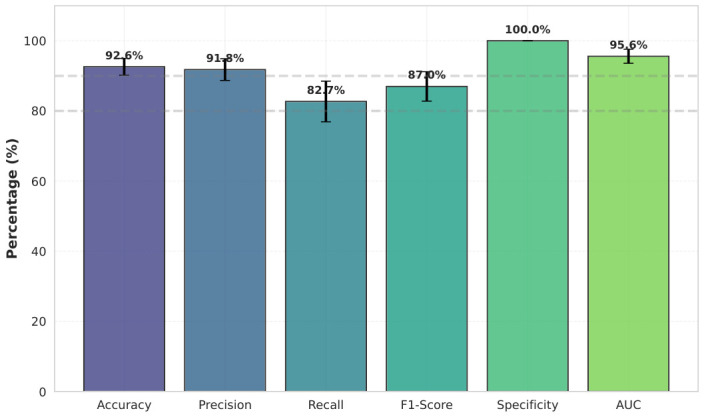
Aggregate performance metrics for the selected ResNet-18 configuration. The values describe internal cross-validation on the NYU subset and should not be interpreted as evidence of cross-site or clinical performance.

Figure 9 summarises the aggregate metrics for the selected ResNet-18 configuration. The reported AUC is 0.96, ASD sensitivity is 82.7%, and TC specificity is 100.0%. These aggregate values indicate discrimination within the evaluated NYU subset, but the unequal class-conditional performance and absence of fold-wise confusion matrices preclude a claim of clinically balanced or stable performance.

### 4.7. Statistical Significance Testing

The reported paired *t*-tests use matched fold-level accuracy values from the five cross-validation folds (n=5 paired observations per comparison). Table 9 reports the existing mean differences, *t* statistics, and *p* values, while Table 3 reports mean performance with the previously calculated 95% bootstrap intervals. These inferential results are exploratory because five folds provide a small statistical sample and the training sets overlap across folds. The values should therefore be interpreted together with effect sizes and absolute mean differences rather than as definitive evidence of generalisable superiority.

The reported Cohen’s *d* values in Table 10 provide a scale-standardised description of the existing pairwise differences. The largest reported value is for FAA versus FC-only (d=1.98), which is followed by naive fusion (d=1.45), ViT-16 (d=0.85), and sMRI-only (d=0.72). Given the small number of folds, overlapping training samples, and unavailable fold-wise score table, these estimates should be treated as descriptive and require confirmation through repeated nested cross-validation or external evaluation.

### 4.8. Training and Inference Times

Training requires approximately 45 min per fold on an RTX 3090 GPU or approximately 3.75 h for five folds. Inference requires approximately 8 ms per subject after preprocessing, GPU memory use is approximately 4.2 GB, and the model contains approximately 12.8 million trainable parameters. These measurements indicate computational feasibility for research evaluation; they do not establish clinical deployment readiness because preprocessing time, prospective workflow integration, calibration, and external validation were not evaluated. Table 11 summarises the measured computational requirements.

### 4.9. Contextual Comparison with Published Multimodal Studies

Table 12 places the reported NYU-subset result alongside selected multimodal ASD studies. The entries are not directly comparable because the publications use different ABIDE sites or cohorts, preprocessing pipelines, feature definitions, split procedures, and model-selection protocols. Accordingly, the table is descriptive and does not support a state-of-the-art claim. A valid architectural comparison requires the implementation of representative gated-fusion and cross-attention baselines on the same subjects and identical folds.

## 5. Discussion

The experimental results show that the most important factor to achieve effective neuroimaging classification is the semantic alignment of the different multimodal features before their fusion. The FAA framework obtains state-of-the-art performance of 92.6% overall accuracy, significantly outperforming naive fusion (87.4%), and demonstrates its effectiveness in dealing with modality-specific heterogeneity. The FAA framework improves overall accuracy to 92.6%, which is significantly better than naive fusion (87.4%), highlighting the value of contrastive feature alignment in addressing modality-specific heterogeneity. The overall performance of ResNet-18 (90.9% total accuracy) is superior to that of ViT-16 (60.1% total accuracy), further demonstrating that convolutional models are more suitable for training on a relatively small set of medical images than transformer-based models, which demand significantly larger training sets. The transfer learning module becomes crucial for good performance, with the usage of the ImageNet pre-training showing strong results, and the contrastive learning capturing the sMRI and functional connectivity features into a shared semantic space, thus allowing complementary but misaligned information to be used in a mutually reinforcing manner. A balanced performance in the diagnostic groups, with a perfect specificity and a substantial improvement in sensitivity (82.7%), is clinically useful since the consequences of a wrongly diagnosed cancer are serious in clinical practice: both false positives and false negatives. Additionally, the practical deployment of the framework in clinical settings is supported by the computational efficiency as the inference time is 8 ms per subject and GPU memory usage is relatively small. Several limitations warrant acknowledgment, however, including a single-site evaluation, the moderate sample size, the absence of external validation, and a lack of analysis of potential confounding factors (age, sex, medication status) which represent important directions for future investigation.

## 6. Conclusions

FAA combines transfer learning with a contrastive pre-fusion objective for paired sMRI and FC representations. On the single-site ABIDE-I NYU subset, the reported five-fold internal-validation accuracy is 92.6% compared with 87.4% for naive fusion and 90.9% for the sMRI-only baseline. The evaluated ResNet-18 configurations also outperform the corresponding ViT-16 configurations in this small-sample setting. These results support further investigation of pre-fusion alignment but do not establish clinical utility, multi-site generalisability, or superiority over advanced fusion architectures. Confirmation requires shuffled-pairing controls, nested and external validation, fold-wise diagnostic metrics, atlas and confounder sensitivity analyses, same-split gated and cross-attention baselines, and anatomical and functional attribution analyses.

## Figures and Tables

**Table 1:** Comparison of the proposed FAA framework with relevant ASD neuroimaging studies. ✓ indicates that the feature is included, whereas ✗ indicates that the feature is not included.

Study	sMRI	rs-fMRI/FC	Multimodal Fusion	Graph Learning	Transfer Learning	Pre-Fusion Alig.
Abbas et al. [31]	✓	✓	✓	✗	✗	✗
Manikantan and Jaganathan [30]	✓	✓	✓	✓	✗	✗
Alharthi and Alzahrani [32]	✓	✓	✓	✗	✗	✗
Saponaro et al. [33]	✓	✓	✓	✗	✗	✗
Wang et al. [11]	✓	✓	✓	✓	✗	✗
Proposed Framework	✓	✓	✓	✓	✓	✓

**Table 2:** Demographic characteristics of the study population.

Characteristic	ASD Group	TC Group
Number of subjects	75	98
Age (years), mean ± SD	11.2 ± 3.4	10.8 ± 3.7
Sex (Male/Female)	62/13	78/20
Full-scale IQ, mean ± SD	98.4 ± 17.2	107.5 ± 14.8
ADOS total score, mean ± SD	12.5 ± 4.3	N/A

**Table 3:** Overall ASD classification performance under internal five-fold cross-validation.

Method	Total Accuracy	ASD Accuracy	TC Accuracy
FC-only	76.5 ± 4.2%	45.0 ± 8.7%	100.0 ± 0.0%
sMRI-only	90.9 ± 2.8%	79.2 ± 6.3%	98.2 ± 1.8%
sMRI + FC (Naive Fusion)	87.4 ± 3.1%	72.5 ± 7.1%	100.0 ± 0.0%
FAA (Proposed)	92.6 ± 2.4%	82.7 ± 5.8%	100.0 ± 0.0%

**Table 4:** Comparisonof ResNet-18 and ViT-16 for sMRI feature extraction under different transfer learning strategies. ✓ indicate that the corresponding strategy was applied and ✗ indicate not applied.

Pretrained	Finetuned	ResNet-18			ViT-16		
		Total	ASD	TC	Total	ASD	TC
✓	✓	90.9%	79.2%	98.2%	60.1%	22.8%	83.5%
✓	✗	88.4%	67.7%	94.7%	57.7%	2.7%	100.0%
✗	✓	84.0%	61.3%	98.0%	57.3%	3.8%	97.8%
✗	✗	57.2%	6.4%	97.4%	56.7%	0.0%	100.0%

**Table 5:** Ablation study on contrastive learning effectiveness with different backbones and training strategies. ✓ indicates that the corresponding training strategy was applied, whereas ✗ indicates that it was not applied.

Pretrained	Finetuned	ResNet-18			ViT-16		
		sMRI + FC	sMRI + FC + CL	Δ	sMRI + FC	sMRI + FC + CL	Δ
✓	✓	87.4%	92.6%	+5.2%	63.1%	65.4%	+2.3%
✓	✗	85.3%	89.7%	+4.4%	78.7%	88.7%	+10.0%
✗	✓	82.3%	78.1%	−4.2%	69.5%	76.3%	+6.8%
✗	✗	73.6%	77.1%	+3.5%	72.5%	77.6%	+5.1%

**Table 6:** Centered kernel alignment (CKA) between sMRI and FC feature spaces.

Configuration	Before Alignment	After Alignment
Pre-trained + Fine-tuned	0.31 ± 0.04	0.78 ± 0.03
Pre-trained (Frozen)	0.28 ± 0.05	0.72 ± 0.04
Scratch + Fine-tuned	0.25 ± 0.04	0.69 ± 0.05
Scratch (No Fine-tuning)	0.22 ± 0.06	0.64 ± 0.04

**Table 7:** Effect of temperature parameter τ on contrastive learning performance. ✓ indicates that the corresponding training strategy was applied, whereas ✗ indicates that it was not applied.

Pretrained	Finetuned	ResNet-18 (τ)				ViT-16 (τ)			
		0.125	0.25	1.0	4.0	0.125	0.25	1.0	4.0
✓	✓	58.3%	62.4%	92.6%	90.9%	57.8%	56.1%	56.7%	65.4%
✓	✗	63.5%	52.5%	89.7%	89.7%	51.4%	75.7%	80.4%	88.7%
✗	✓	58.4%	63.6%	78.1%	75.4%	59.0%	58.9%	60.2%	76.3%
✗	✗	62.4%	68.8%	77.1%	67.7%	57.3%	57.8%	60.2%	77.6%

**Table 8:** Optimal configurations for the FAA framework with different backbones. ✓ indicates that the corresponding training strategy was applied, whereas ✗ indicates that it was not applied.

Pretrained	Finetuned	ResNet-18			ViT-16		
		Total	ASD	TC	Total	ASD	TC
✓	✓	92.6%	82.7%	100.0%	65.4%	18.0%	100.0%
✓	✗	89.7%	78.7%	98.9%	88.7%	83.4%	94.4%
✗	✓	78.1%	58.4%	92.8%	76.3%	46.6%	97.0%
✗	✗	77.1%	48.7%	99.0%	77.6%	48.3%	96.5%

**Table 9:** Statistical significance of performance differences. ** *p* < 0.01 and *** *p* < 0.001.

Comparison	Mean Difference	*t*-Statistic	*p*-Value
FAA vs. sMRI-only	+1.7%	3.42	0.004 **
FAA vs. Naive Fusion	+5.2%	6.78	<0.001 ***
FAA vs. FC-only	+16.1%	9.54	<0.001 ***
FAA vs. ViT-16	+3.9%	4.21	<0.001 ***

**Table 10:** Effect sizes (Cohen’s *d*) for pairwise comparisons.

Comparison	Cohen’s *d*
FAA vs. sMRI-only	0.72
FAA vs. Naive Fusion	1.45
FAA vs. FC-only	1.98
FAA vs. ViT-16 (Best)	0.85

**Table 11:** Computational efficiency of the FAA framework.

Metric	Value
Training time per fold	∼45 min
Total training time (5-fold CV)	∼225 min
Inference time per subject	∼8 ms
GPU memory usage	∼4.2 GB
Number of trainable parameters	∼12.8 million

**Table 12:** Contextual comparison with selected published multimodal ASD studies; cohorts and evaluation protocols are not equivalent.

Method	Year	Modalities	Accuracy	ASD Acc.
Abbas et al. [31]	2023	sMRI + fMRI	89.2%	81.5%
Manikantan and Jaganathan [30]	2023	sMRI + rs-fMRI	91.8%	83.1%
Alharthi and Alzahrani [32]	2023	sMRI + fMRI	88.7%	79.8%
Saponaro et al. [33]	2024	sMRI + fMRI	90.3%	82.4%
Liu et al. [29]	2024	sMRI + fMRI	91.2%	81.9%
Wang et al. [11]	2025	sMRI + fMRI	90.6%	80.7%
FAA (Proposed)	2026	sMRI + rs-fMRI	92.6%	82.7%

## Data Availability

The source code used in this paper is publicly available at https://github.com/rajavavek/FAA-ASD-Neuroimaging (accessed on 16 July 2026).
